# Imaging Atherosclerosis with Hybrid Positron Emission Tomography/Magnetic Resonance Imaging

**DOI:** 10.1155/2015/914516

**Published:** 2015-01-28

**Authors:** Rasmus Sejersten Ripa, Andreas Kjær

**Affiliations:** Department of Clinical Physiology, Nuclear Medicine & PET, Rigshospitalet & Cluster for Molecular Imaging, Faculty of Health and Medical Sciences, University of Copenhagen, KF-4012 Rigshospitalet, Blegdamsvej 9, 2100 Copenhagen, Denmark

## Abstract

Noninvasive imaging of atherosclerosis could potentially move patient management towards individualized triage, treatment, and followup. The newly introduced combined positron emission tomography (PET) and magnetic resonance imaging (MRI) system could emerge as a key player in this context. Both PET and MRI have previously been used for imaging plaque morphology and function: however, the combination of the two methods may offer new synergistic opportunities. Here, we will give a short summary of current relevant clinical applications of PET and MRI in the setting of atherosclerosis. Additionally, our initial experiences with simultaneous PET/MRI for atherosclerosis imaging are presented. Finally, future potential vascular applications exploiting the unique combination of PET and MRI will be discussed.

## 1. Introduction

Molecular imaging can be defined as noninvasive visualization and quantification of distinct molecular pathways. Important features of molecular imaging are the ability to target a molecular process in living organisms without perturbing them. Both positron emission tomography (PET) and magnetic resonance imaging (MRI) are classic methods used for molecular imaging.

Atherosclerosis is traditionally assessed using luminal stenosis by anatomical imaging such as angiography or ultrasound. However, atherosclerosis is now recognized as a systemic degenerative inflammatory vascular disease that develops over decades, with a long subclinical period. Postmortem analyses have shown that most fatal vascular events originate from nonstenotic atherosclerotic lesions [1], and about half of all patients who die from coronary heart disease have no prior diagnosis or symptoms of cardiac disease [2]. Despite this fact, screening asymptomatic adults for cardiovascular risk by imaging is considered inappropriate in most cases by current guidelines [3].

The concept of the vulnerable plaque is a hallmark in atherosclerosis. The vulnerable atherosclerotic plaques are those with a high short-term risk of rupture and thrombosis. The vulnerability of a plaque is characterized by a number of factors like a thin, collagen-poor fibrous cap, a large necrotic core, and abundant macrophages in the cap, whereas the luminal protrusion is not a marker of vulnerability [4].

The current goal in noninvasive imaging is to identify vulnerable atherosclerotic plaques that may subsequently lead to myocardial infarction or stroke. This identification could lead to more optimal and individualized risk stratification and thereby enabling personalized therapy.

The aim of this review is to give a summary of current relevant clinical applications of PET and MRI in the setting of atherosclerosis and to discuss potential future uses of the newly introduced combined PET/MRI system.

## 2. Why Hybrid Imaging with PET and MRI?

**Figure 1:**
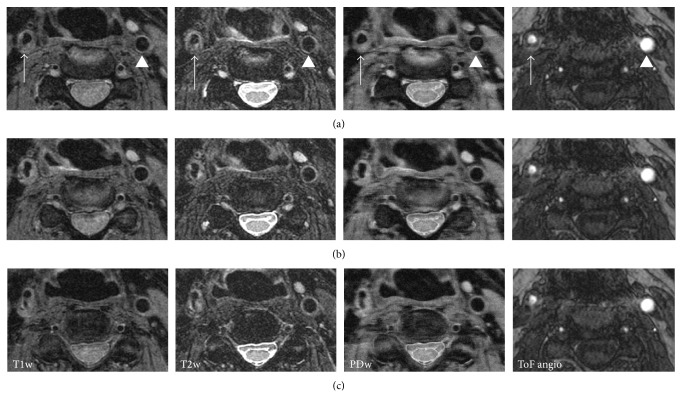
Example of multisequence MRI of a plaque in the right carotid artery (arrow). Each column shows three slices from the right common carotid artery (top row) to the internal carotid artery (bottom row). The nonstenotic left coronary artery is shown for comparison (arrowhead). Four different MR sequences are shown (T1 weighted, T2 weighted, proton density weighting, and time-of-flight angiography).

A state-of-the-art MRI scanner offers the ability to perform both anatomical and functional examinations. The atherosclerotic plaque components can be differentiated using dedicated imaging sequences (Figure 1). T1-, T2-, and proton density weighted imaging of carotid plaques allows for identification of the lipid-rich necrotic core, calcification, and intraplaque hemorrhage. The high spatial resolution of MRI even allows for identification and assessment of the fibrous cap. One study published as early as 2002 included 60 patients scheduled for carotid endarterectomy. The carotid arteries were imaged in vivo with a 1.5-T scanner (time of flight and T1-, PD-, and T2-weighted). The plaque classification from this multisequence MRI showed good agreement with the American Heart Association classifications from the subsequent histological examination (Cohen's kappa of 0.74) [5]. Since then, the imaging technique has been improved by optimization of the image sequences used, the introduction of new imaging sequences, and increased magnetic field strength in the MRI system. The fibrous cap is a major contributor to the vulnerability of the plaque. The feasibility of fibrous cap visualization by MRI in the carotid artery is well established [6, 7], and fibrous cap rupture is associated with cerebrovascular symptoms in both prospective and cross-sectional studies [8, 9]. Neoangiogenesis in the atherosclerotic plaque is also considered a hallmark of the unstable plaque. Some studies indicate that dynamic contrast enhanced MRI utilizing gadolinium-based extracellular contrast agents can be used to assess microvessel density in the plaques [10].

MRI for plaque characterization in the coronary arteries is technically more challenging than carotid plaques due to cardiac and respiratory motion of the often small tortuous vessels. Several methods are under development to deal with these challenges. Some studies have in fact shown that positive remodeling and intracoronary thrombus detection is feasible in the coronary arteries [11, 12].

Molecular imaging with MRI is also possible using specific contrast agents that allow for visualization of processes in the atherosclerotic plaque at the molecular level. Target specific MRI contrast agents are typically based on paramagnetic gadolinium or iron oxide. Clinical studies have demonstrated uptake of small iron oxide (USPIO) particles in carotid plaques and the uptake was found to correspond to areas of macrophage infiltration [13]. One study has even used USPIO-enhanced carotid MRI to assess the therapeutic response of short term aggressive lipid lower therapy [14]. Fibrin is another molecular target of MRI utilizing a fibrin-specific gadolinium-based contrast agent. This agent has been used in a few clinical studies for thrombi detection [15]. A number of other target specific MRI contrast agents for imaging atherosclerosis are currently being tested in animal models (review in [16]).

It is relevant in this context to consider what PET can add to the “MRI-one-stop-shop.” Clinical PET has a spatial resolution around 3 mm at best and can thus in no way compete with the spatial resolution of MRI. PET is based on photon emission from positron emitting radioactive tracers and does not contain anatomical information. Thus, PET cannot be used to image morphological components of the plaque. PET, however, is a sublime technique for molecular imaging. Molecular imaging with MRI has a limited sensitivity typically in the micromolar range, whereas a typical clinical PET scanner can detect concentrations in nano- to picomoles per liter. This sensitivity, however, is lower in plaque imaging since the plaque size is near the spatial resolution of the system and thus subject to signal loss by partial volume effects.

PET is based on the tracer principle for imaging. The radioactive tracer utilized in PET is a compound where an atom is replaced by a radioisotope or a radioisotope is added. Only traces of the substance are applied; therefore, it has no pharmacologic effect in vivo. Typically, the tracer is a biomolecule that reflects a particular body function or metabolism. PET tracers can be nonspecific following a biochemical pathway or allowing for measurement of tissue extraction or metabolism. These radiotracers include the glucose analogue fluorine-18-fluorodeoxyglucose (FDG) which is taken up by high-glucose-utilizing cells, where FDG is trapped intracellularly by phosphorylation to allow tissue glucose metabolism assessment. PET tracers can also be specific radioligands involved in an interaction with receptors. PET tracer distribution can be quantified in absolute terms and with dynamic measurement of PET tracer uptake and distribution kinetic analysis is possible.

In summary, we find that molecular imaging with PET in most cases will be far superior to molecular imaging with MRI. In our opinion, optimal utilization of the complementary information from hybrid PET/MR system will require a better understanding and foremost a better use of the pathophysiologic information that can be acquired by MRI. Simply using the anatomical information in conjunction with the metabolic information from the PET will be a suboptimal utilization of the potential of the system.

## 3. Initial Clinical Experience

### 3.1. Large Animal Model

**Figure 2:**
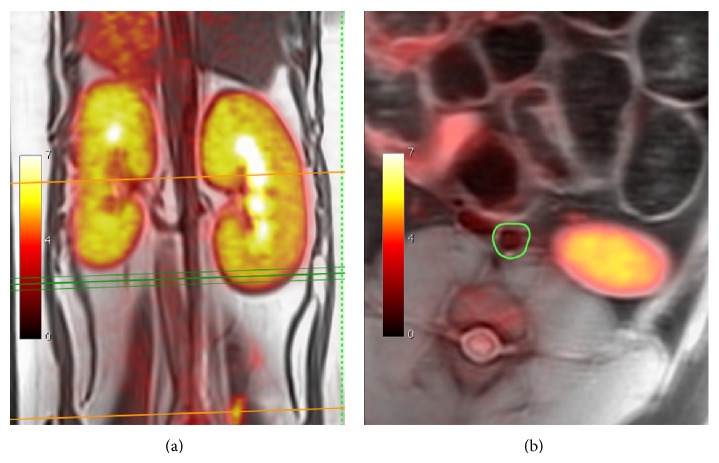
Example of vascular simultaneous FDG-PET/MRI from porcine model. The abdominal aorta is outlined in green (reproduced from [18] with permission from the editor).

As compared to preclinical animal scanners, human hybrid PET/MRI systems can physically contain larger animals. This opens the opportunity to study atherosclerosis in human-like atherosclerosis models such as rabbits and porcines. So far, few feasibility data on this topic has been published; Dregely et al. [17] presented feasibility data from 4 high-cholesterol fed rabbits as an abstract at the 2012 annual meeting of Society of Nuclear Medicine and Molecular Imaging. Likewise, our institution is working with a porcine model of atherosclerosis (Figure 2). At our hand, simultaneous PET/MRI of this large animal model is feasible but requires some optimization [18].

### 3.2. Vascular Imaging in Humans without Atherosclerosis

**Figure 3:**
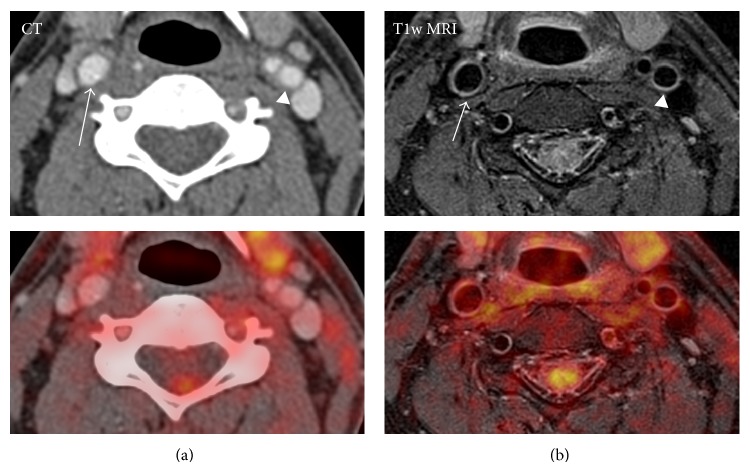
Comparison of contrast enhanced CT (a) with T1 weighted MRI (b) for vessel delineation in patients without significant carotid plaque. The right common carotid artery (arrow) and left internal carotid artery (arrowhead) are shown. The bottom row shows fusion with FDG-PET. This patient was part of a previous published trial [19].

Our institution recently performed a study aiming at evaluating the feasibility of integrated PET/MR imaging of the carotid arteries in humans [19]. Six HIV patients with increased risk of atherosclerosis but without any symptoms of cardiovascular disease were included to a single-FDG-injection dual-imaging protocol of simultaneous PET/MR and subsequent integrated PET/CT on the same day. It is clear from Figure 3 that MR allowed for superior delineation of both the inner and outer walls of the carotid artery as compared to the CT in the study. The study found a high congruence between FDG-uptake quantification using the two systems despite the inherent methodological differences between the two systems such as method of attenuation correction, the use of time-of-flight in PET, and the potential interference of the MR signal from PET detectors inside the MR scanner.

### 3.3. PET/MRI of Human Atherosclerosis in the Carotid Arteries

**Figure 4:**
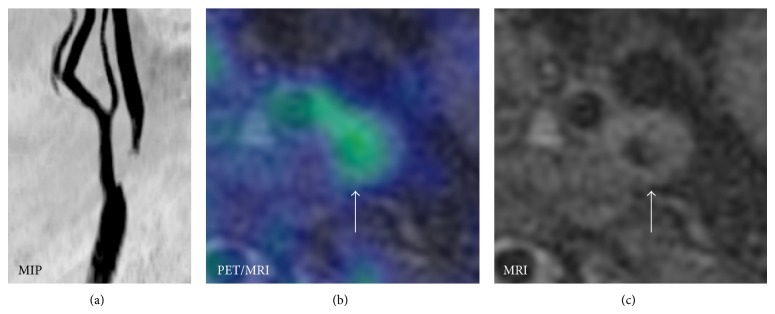
Example of simultaneous acquisition FDG-PET and MRI using biograph mMR. (a) MR angiography with severe proximal stenosis of left internal carotid artery. (b) Fusion of FDG-PET (color) and MRI with increased FDG-uptake in the internal carotid artery. (c) Transverse MR showing stenosis of internal carotid artery.

PET imaging of atherosclerosis has so far focused primarily on FDG. The first report on FDG accumulation in the large arteries emerged in 2001 [20], and since then, a large body of evidence has materialized linking FDG uptake to the macrophage contents of high-risk plaques [21–23]. The idea of combining FDG-PET with MRI is not new. In fact several studies have used sequential PET and MRI for imaging atherosclerotic plaques in animal models [24] as well as in carotid plaques in humans [25–29]. In our experience carotid plaque with simultaneous FDG-PET/MRI is feasible (Figure 4). Future studies will have to show if FDG-PET/MRI has superior prognostic information as compared to FDG-PET/CT.

One very interesting trial is the ongoing prospective observational PESA study [30]. A subgroup of 1,300 participants within this study which have evidence of atherosclerosis on ultrasound or increased coronary artery calcium score will undergo hybrid FDG PET/MRI study of both the carotid and iliofemoral arteries and the FDG PET/MRI study will be repeated at 6 years of followup.

## 4. Potential Vascular PET/MRI Applications

### 4.1. New PET Tracers

PET imaging of atherosclerosis has thus far focused primarily on FDG. A major drawback of imaging atherosclerosis with FDG PET, however, is the lack of specificity of the tracer. Another limitation is the high uptake of FDG in the myocardium, which produces a suboptimal signal-to-noise ratio when coronary arteries are imaged. As an alternative, some researchers have suggested 18F-deoxy-mannose as a more atherosclerosis specific tracer than FDG [31], but this needs to be confirmed in clinical trials.

A hunt for new and more specific tracers has started. The tracers should specifically target cell-mediated key molecular processes associated with the vulnerable atherosclerotic plaque. The most prominent of these targets include macrophage infiltration, apoptosis, hypoxia, and neoangiogenesis of the intima/media. Activated macrophages express the somatostatin receptor subtype 2, and this could be a target for PET imaging. ^68^Ga-DOTATATE is utilized in diagnosis and staging of neuroendocrine tumors and has high affinity for somatostatin receptors and a few studies have suggested a future role in plaque imaging [32–34]. PET tracers for imaging apoptosis, hypoxia, and neoangiogenesis are available, but their use in imaging atherosclerosis is very limited thus far [35–37].

Another promising PET tracer for plaque imaging is ^18^F-sodium fluoride (NaF). This tracer is deposited by chemisorptions onto hydroxyapatite and is used in oncology to identify bone metastasis. Recent evidence suggests that NaF uptake is not equivalent to calcification as identified by CT imaging [38] but that it can identify “spotty” metabolic active calcification in the plaques thought to promote plaque vulnerability. Using hybrid PET/CT and a retrospective approach, it was recently described how NaF accumulated in atheroma of the aorta, iliac, femoral, and carotid arteries [39]; however, coincidental NaF and FDG uptake (14 of 215 lesions) is rare [40]. Joshi et al. [41] recently published an interesting study indicating that NaF PET can identify culprit and ruptured plaques in patients with recent myocardial infarction.

In summary, we find that the combination of morphologic and functional information from the MRI with molecular imaging from PET may lead to optimal characterization of the plaque and thus improved individualized counseling and therapy.

### 4.2. Spectroscopy and Hyperpolarization

Image-guided proton magnetic resonance spectroscopy (^1^H-MRS) of atherosclerotic plaques in carotid arteries using clinical 3-T MR systems is feasible [42]. The proton spectrum is collected from image-localized plaques, so that the specific proton resonances can be identified. Duivenvoorden et al. [42] have recently used this method to identify liquid phase cholesteryl ester in carotid plaques. The trial was not without challenges though. The proton spectra were collected from a voxel of 5 × 5 × 5 mm with 13 minutes acquisition time. Only 49% of the obtained spectra were of adequate quality for analysis. No studies combining proton spectroscopy with PET have so far been published in the field of atherosclerosis.

Our institution has recently installed a hyperpolarizer with our PET/MRI system allowing us to do simultaneous hyperpolarized MR and PET (HyperPET). Hyperpolarization of nuclear spins like ^13^C can increase the MR signal with a factor of more than 10^5^. Chemical compounds (tracers) like pyruvate can be enriched with ^13^C and injected into humans or animals following ex vivo hyperpolarization. The increased signal from the ^13^C allows both imaging and spectroscopy of the tracer within a limited time window. Hyperpolarized MRI for atherosclerosis has only been reported in few preclinical reports [43]. The HyperPET offers several new applications for in vivo molecular imaging of atherosclerosis. Due to its physical nature, PET can only image one radioactive tracer at the time. In comparison, HyperPET can measure several biological processes simultaneously; for example, plaque glycolysis can be assessed with hyperpolarized ^13^C-pyruvate and plaque hypoxia can be assessed with a specific radiolabelled PET tracer like ^64^Cu-ATSM simultaneously. In this way, HyperPET can combine two molecular imaging techniques with a possible synergistic effect in atherosclerosis. HyperPET, however, is a very demanding and expensive solution that will most likely be restricted to experimental use in few selected cases. Nevertheless, the distinct and small volume of interest, the plaque, makes HyperPET more realistic to be applied than in diseases where whole body evaluation is needed.

### 4.3. Research Platform

**Figure 5:**
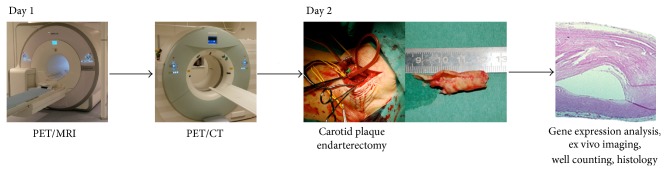
Example of multimodality imaging workflow from our institution.

The acquisition of a PET/MRI system is a huge task that requires widespread knowhow by technicians, physicists, and physicians in addition to extensive funding both initially and for running costs. It is our expectation that this will limit the technique to mainly experimental use in a limited number of major university centers for some time. At our institution, we have set up an atherosclerosis research workflow in collaboration with the vascular surgeons (Figure 5). This workflow allows our staff technicians to become familiar with the multimodality setup, but at the same time gives us flexibility to introduce new imaging sequences, reconstructions, or ex vivo molecular techniques.

One apparent area for simultaneous PET/MRI is cross validation of new imaging modalities when similar molecular imaging probes exist in PET and MRI. The simultaneous acquisition allows for experiments to be performed under one and the same physiologic condition. One very elegant example is the use of a reporter gene approach. Higuchi et al. [44] transduced endothelial progenitor cells with the sodium iodide symporter gene for reporter gene imaging by PET and also labeled the cells with iron oxides for visualization by MRI. After intramyocardial injection, cells were followed with both PET and MRI. The PET tracer uptake decreased and was undetectable on day 7, whereas the MRI signal remained unchanged throughout the follow-up period. Histological analysis confirmed the presence of labeled transplanted cells at the site on day 1 but not on day 7, when only iron-loaded macrophages were seen [44]. This example clearly states the difference between anatomical imaging with MRI and molecular imaging with PET.

### 4.4. Interventional Studies of Atherosclerosis

Both PET and MRI have been used for followup after clinical intervention. The dal-PLAQUE study randomly assigned 130 patients with, or with high risk of, coronary heart disease to placebo or dalcetrapib [45]. Coprimary endpoints were MRI-assessed structural changes in the arterial wall after 24 months and assessment of arterial inflammation with FDG PET/CT. Also, some animal studies have utilized sequential PET/CT and MRI as endpoint in interventional trials [46, 47]. There are, however, clear advantages of using simultaneous PET/MRI as compared to PET/CT and stand-alone MRI in this setting. The simultaneous acquisition diminishes the problem with correct alignment between sequential examinations; this could be of particular importance in a follow-up trial where the expected plaque changes might be small. The use of repetitive CT examinations inflicts a nonnegligible radiation dose to the patients that is avoided when using MRI.

To date, no interventional studies of human atherosclerosis using hybrid PET/MRI have been published.

## 5. Conclusion

We have presented our initial experiences with simultaneous PET/MRI in the field of atherosclerosis. It is our belief that the synergy between PET and MRI will justify its use in atherosclerotic imaging despite its higher cost and more complex management. At the same time though, we do not see a translation from experimental applications to clinical routine in the very near future.

We expect a continuous development of the integration of molecular, functional, and anatomical imaging as well as the clinical indications within atherosclerosis. This novel imaging methods may lead to early detection of high-risk vulnerable plaques, enabling clinicians to improve risk stratification and thus paving the way for individualized therapy.
